# Environmental sustainability, medical waste management, energy and medicine consumption of the surgical intensive care nurses: A qualitative study

**DOI:** 10.1111/nicc.13150

**Published:** 2024-08-15

**Authors:** Yeliz Sürme, Gülseren Maraş, Gökçen Aydin Akbuğa

**Affiliations:** ^1^ Surgery Nursing, Faculty of Health Sciences Erciyes University Kayseri Turkey; ^2^ Surgery Nursing, Faculty of Health Sciences Yozgat Bozok University Yozgat Turkey

**Keywords:** energy, environmental sustainability, intensive care nursing, medicine, waste management

## Abstract

**Background:**

In intensive care units, it is noticeable that there is intensive use of resources in the treatment and care process, leading to a significant amount of waste generation. In addition, the demand for intensive care, increasing life expectancy and surgical interventions, complex comorbidities and ecological crisis make it necessary to make critical care more sustainable.

**Aim:**

To explore the perspectives of nurses working in surgical intensive care units regarding responsible medical waste management, energy and medication consumption.

**Study Design:**

This qualitative descriptive study was conducted in surgical intensive care units of a university hospital in Turkey in November 2023. Twenty‐three nurses filled in an introductory form and participated in a semi‐structured interview. Data were analysed using inductive content analysis.

**Results:**

Three main themes were determined: environmentally sustainable intensive care, prevention of waste in intensive care; responsible consumption and recycling; suggestions for institutional and individual behavioural change regarding environmental sustainability.

**Conclusions:**

The majority of nurses lack knowledge about sustainable development goals. However, in the intensive care unit, they provided effective and creative solutions for medical waste management, energy and medication consumption and individual and institutional behavioural change regarding environmental sustainability.

**Relevance to Clinical Practice:**

Sustainability strategies should be created in institutions to ensure responsible medical waste management, energy and medicine consumption and reduce carbon footprint. In accordance with this purpose, ‘Green teams’ including unit‐based doctors, nurses and paramedics should be established. Training should be provided and awareness should be raised to reduce energy use resulting from heating, lighting, ventilation and air conditioning.

**What is known about the topic**

- Intensive care units consume large amounts of resources and generate high volumes of waste.
- Previous studies have focused on the amount of medical waste generated in intensive care units.

**What this paper adds**

- Intensive care nurses have the potential to greatly contribute to improving environmental sustainability.
- Intensive care nurses view training, reward and control as three main elements for minimizing waste from energy usage and consumables.
- Sustainability requires a collaborative team effort, including nurses, doctors, pharmacists, allied health professionals, hospital management, estates and engineers all working together to reduce intensive care's environmental impact.

## 1 INTRODUCTION

Environmentally sustainable health care is a broad concept that encompasses the economic, environmental and social dimensions to ensure the efficient delivery of health care services, the effective use of resources and the continuity of health care for future generations. Health care services significantly consume many resources such as water, electricity, medications, food and consumables, and they also pose a potential source of harm in terms of waste and carbon footprint.^1^, ^2^ The health care sector is responsible for approximately 4%–5% of global greenhouse gas emissions.^3^ In the United States, the carbon footprint of the health care system constitutes about 9%–10% of national greenhouse gas emissions.^4^, ^5^ Similarly, in Australia and European countries like the United Kingdom and the Netherlands, it is approximately 7%.^4^ In 2019, the carbon footprint of the United Kingdom's National Health Service (NHS) reached a total of 25.0 megatons CO_2_ equivalent (CO_2_e). Of this, 62% originated from the supply chain, 24% from the provision of care, 10% from staff commuting, patient and visitor travel and 4% from private health and care services commissioned by the NHS.^3^ In a hospital in Germany, it was reported that in 2019, daily emissions per patient amounted to 157 kg CO_2_e per bed ranged from 34.400 to 36.900 kg CO_2_e and per full‐time equivalent staff member ranged from 20 700 to 22.200 kg CO_2_e. The most significant sources of emissions were the production and use of heating fuel (40%–43%), the production of medical supplies (19%–25%) and electricity (13%–14%).^6^

From a sustainability perspective, intensive care units (ICUs) have become significant areas because of high resource consumption, large amounts of waste, increased life expectancy, surgical interventions and the growing need for care resulting from complex comorbidities.^2^, ^7^ A study^8^ conducted in an ICU in the Netherlands aimed to analyse the material flow, assess the environmental footprint of materials and identify significant environmental impact points. The study found that the environmental impact per patient in the ICU resulted in 17 kg of mass, 12 kg CO₂ equivalent, 300 L of water use and the occupation of 4 m^2^ of agricultural land per day. Critical materials included non‐sterile gloves, isolation gowns, bed linens, surgical masks and injectors.^8^ Transitioning energy sources from coal and natural gas to renewable sources and opting for sustainable rather than single‐use materials could have the greatest impact on reducing the ICU's carbon footprint. This approach could effectively lower the carbon footprint regardless of the intensity of care provided.^2^

The World Health Organization (WHO)^9^ has published a report for its stakeholders on eliminating the harmful effects of health care services and providing sustainable and climate‐friendly health care services. The report stated that the health care sector has a leading role in providing sustainable service globally and identified seven themes for climate‐friendly hospitals. These include (1) reducing energy consumption, (2) alternative energy sources, (3) disseminating green sustainable health buildings, (4) encouraging public transportation, (5) sustainable food, (6) waste management and (7) water saving. Besides, the International Council of Nurses^10^ emphasized the common responsibility of nursing to protect and maintain the natural environment from depletion and pollution, and recommended that nurses who provide uninterrupted care services should be aware of sustainability.

Considering reports and recommendations, it is evident that nurses have immense potential in tackling waste management and in reducing the environmental footprint and costs of the health care sector. Ensuring sustainability in nursing care, responding to climate change and providing solutions in this context first require understanding the concept of sustainable intensive care and investigating nurses' perspectives on this issue.^11^, ^12^ A qualitative study^13^ conducted with intensive care nurses in the United Kingdom highlighted their concerns regarding the current resource utilization model in critical care units, which they deemed unsustainable for the future in economic, ecological or social terms. The study emphasized that sustainability is related to the decision‐making processes regarding whether and how resources are used during the care of critically ill patients.^13^

Research explaining what sustainability means for nurses working in intensive care and how it affects their practices is limited. Understanding these perspectives is crucial for implementing effective sustainability measures in critical care settings. Therefore, this study was conducted with the aim of revealing nurses' views on environmentally sustainable intensive care.

## 2 METHOD

### 2.1 Type of research

This study was designed as a qualitative descriptive and interview study. This design allowed us to deeply understand the perspectives of intensive care nurses regarding environmentally sustainable intensive care. A qualitative descriptive design is intended to describe rich and realistic perspectives of individuals who have experienced a specific and focused situation.^14^ Qualitative description (QD) is based on a direct and rich description of experiences or events and presents data in the clearest way without straying into extensive theorizing or abstractions.^15^ QD is an important method for research questions that focus on discovering the who, what and where of events or experiences and gaining insight from informants into a poorly understood event.^16^

### 2.2 Study population

The study was conducted in November 2023 with nurses working in the surgical ICU of a university hospital located in the Central Anatolia region of Turkey. In the tertiary hospital of the province where the study was conducted, there is one surgical ICU. Across Turkey, there are 70 university hospitals providing tertiary health care services (7020 ICU beds).^17^ The population of the study consists of 23 nurses working in surgical ICUs, having a bachelor's degree, volunteering to participate in the study. Purposive sampling method was used. As it is a qualitative study, a clear sample number has not been determined as stated in the literature.^18^ Therefore, when the data started to repeat, it was thought that data saturation was reached. The study was completed with 23 nurses, ensuring data saturation.

### 2.3 Data collection

Data were obtained using an introductory information form and a semi‐structured interview form. Quantitative data such as age, gender and working years, the most commonly used consumable material and natural resource in the clinic, were filled in by the participants using an introduction form before commencement of semi‐structured interviews for collecting qualitative data. The semi‐structured interview form was developed by the authors based on the related literature and consisted of six open‐ended questions^19^ (Table 1).

**TABLE 1:** Semi‐structured interview questions.

1.	What do environmentally sustainable intensive care and responsible consumption mean to you?
2.	Do you have any initiatives regarding the sustainable use of personal protective equipment? What are your recommendations in this regard?
3.	What are the obstacles to the proper management of medical waste and recycling in the department where you work? What measures can be taken to ensure appropriate medical waste management?
4.	Do you have any initiatives regarding responsible medication use/consumption? What are your recommendations in this regard?
5.	Do you have any initiatives related to sustainable energy use (electricity, water, natural gas) and responsible energy consumption? What recommendations do you have in this regard?
6.	What are the obstacles to creating both institutional and individual behavioural change regarding environmental sustainability? What measures can be taken to facilitate behavioural change in this context?

The interview setting was a quiet environment, free from noise or other distracting elements, where both the researcher and the participant could comfortably engage in conversation. The interviews were conducted in a one‐on‐one, face‐to‐face format. The interviews were conducted by an associate professor in surgical nursing (GAA). Given the author's expertise in the field and understanding of the issues faced by nurses, a conducive atmosphere was created, allowing nurses to communicate comfortably and confidently. In the interviews, with the participants' consent, audio recordings were obtained. The interviews had an average duration of 30–35 min. The questions were posed in the same sequence during the interviews, and additional explanations were provided as needed. The researcher posed follow‐up questions such as ‘Could you provide more detail?’ or ‘Could you clarify this?’ Unintelligible questions and answers were reiterated. Each participant underwent a single interview.

### 2.4 Data analysis

The quantitative data were expressed in terms of numbers, averages, standard deviations and percentages. Qualitative data were analysed through an inductive content analysis.^18^ Inductive content analysis systematically and objectively reveals the way to describe incompletely understood events.^20^ Data analysis was conducted by two researchers (YS, GM).The data were analysed in seven steps: (1) After listening to the audio recordings, the participants' statements were manually transcribed verbatim to ensure a thorough understanding of their expressions. (2) Written documents were read multiple times by two researchers (YS, GM), and significant sections were underlined. (3) Meanings were constructed from key expressions, and significant expressions were determined based on the statements frequently articulated by the nurses. (4) Meanings were grouped, and themes and sub‐themes were formed. For example, education, reward—encouragement, and control are grouped under the main theme of suggestions for institutional and individual behavioural change regarding environmental sustainability. (5) All the themes and sub‐themes generated were comprehensively integrated with the experiences of the nurses. (6) The foundational structure of the nurses' experiences was established. (7) A summary of the interview was prepared with the participants to clarify the results and confirm the meaning of the statements after the interview.

Validity and confirmability of the generated themes and sub‐themes were ensured by obtaining expert opinions from two independent researchers (YS, GM) with qualitative research training and experience. Following the expert opinions, unnecessary repetitions were eliminated, relevant ones were reorganized and the final structure of themes and sub‐themes was established. Themes have been supported with direct quotations.^18^ The reporting of the study adhered to the Consolidated Criteria for Reporting Qualitative Research guidelines.^21^

### 2.5 Ethical statements

The nurses were informed about the study and their written and verbal consents were obtained. Ethical approval was obtained from the Erciyes University Ethics Committee (08/24). The names of participants and relevant data have been kept confidential. (e.g. audio recordings and transcriptions were kept in a secure location, the names of the nurses were removed from all documents and assigned codes [e.g., N1, N2, N3]).

### 2.6 Rigour

The rigour of this study was strengthened based on Colorafi and Evans^22^ strategies for confirmability, dependability, credibility, transferability and application. For confirmability, individuals' statements have been directly incorporated into the research report. Dependability was ensured by having the same researcher conduct all the interviews. For the credibility of the study, a trust relationship was established with the participants at the beginning of the interviews, and they were encouraged to express their opinions through prolonged discussions. Expert opinions were sought regarding whether the sub‐themes under the conceptual theme reached in the study represented the specified conceptual category to ensure the reliability of the research. Transferability and application involve how the results can be generalized and applied to the entire population. In qualitative research, transferability suggests the applicability of the findings from one study to other contexts or groups of people. Determining whether the findings are transferable is possible by explaining contextual factors influencing the research and the methods followed in sample selection.^16^ In this study, sample selection, participant characteristics and the conduct of the study are detailed to assist readers in considering the applicability of the findings to their own situations.

### 2.7 Reflexivity

Reflexivity is acknowledged as a crucial instrument for enhancing the precision and dependability of qualitative research. The criteria for precision and dependability in qualitative studies involve highlighting the relational aspects of knowledge construction through transparency, reciprocity and critical self‐reflection.^23^ The research team consists of three researchers, all of whom are academics. The first is an associate professor in surgical nursing with publications on intensive care, medical waste and recycling. The second is currently pursuing a doctoral degree in surgical nursing and has worked as a perioperative nurse for 7 years. The third is also an associate professor in surgical nursing. The researcher conducting the interview is experienced in qualitative studies, has qualitative publications and has conducted interviews in qualitative studies. She is an associate professor in the field of surgical nursing and has been working as an academician for 15 years. The fact that the researchers are surgical nurses has been a significant factor in maintaining effective communication and trust with the participants. The researchers have substantial experience in nursing research, contributing valuable insights to data processing and interpretation. Working in an independent institution, the researchers may provide increased transparency in participants' responses (Table 2).

**TABLE 2:** Demographic characteristics of the nurses (*n* = 23).

Demographic variable	*n*	%
Gender		
Female	17	73.9
Male	6	26.1
Age (mean ± SD [min–max])	29.17 ± 6.26 (22–50)	
Total working year (mean ± SD [min–max])	6.69 ± 6.22 (1–31)	
Working year in the surgical intensive care (mean ± SD [min–max])	4.14 ± 2.34 (1–9)	
Educational status		
Bachelor's degree	18	78.2
Master's degree	5	21.8
Most commonly used consumables in intensive care^a^		
Personal protective equipment	8	34.7
Medical consumables (injector, intravenous catheter, needle tip, serum set, etc.)	17	73.9
Dressing materials	13	56.5
Catheters (nasogastric, bladder, etc.)	8	34.7
Bed sheets	7	30.4
Paper	3	13.04
Most consumed energy source^a^		
Electric	21	91.3
Natural Gas	1	4.3
Water	4	17.3
Information on sustainable development goals		
Yes	3	13.1
No	20	86.9

^a^ Multiple answers were given.

## 3 RESULTS

In the study, it was determined that 73.9% of the nurses were female, with an average age of 29.17 ± 6.26. Additionally, 78.2% had a bachelor's degree. The nurses' average duration of working in surgical ICU was found to be 4.14 ± 2.34. In terms of consumables most frequently consumed in intensive care, 73.9% of nurses reported the highest consumption of medical supplies such as injector, intravenous catheters and needle tips, ranking first; and 56.5% indicated the second‐highest consumption of dressing materials. Furthermore, 91.3% of nurses reported electricity as the most consumed source of energy in intensive care. It was noted that 86.9% of nurses expressed a lack of awareness regarding sustainable development goals.

As a result of the study, three main themes and nine sub‐themes were determined that revealed the nurses' views on environmentally sustainable intensive care (Table 3).

**TABLE 3:** Themes and sub‐themes.

Themes	Sub‐themes
Theme 1: Environmentally sustainable intensive care	Minimizing, conscious segregation and recycling of medical waste Waste reduction and cost‐effectiveness Leaving a clean environment for the future generations
Theme 2: Prevention of waste in intensive care, responsible consumption and recycling	Drug wastage Energy waste Consumable consumption and recycling
Theme 3: Suggestions for institutional and individual behavioural change regarding environmental sustainability	Education Reward—Encouragement Control

### 3.1 Theme 1: Environmentally sustainable intensive care

Environmentally sustainable intensive care is stated as minimizing medical waste, conscious separation and recycling, reducing waste and cost‐effectiveness, leaving a clean environment for future generations.

#### 3.1.1 Sub‐theme 1.1: Minimizing, conscious segregation and recycling of medical waste

More than half of the nurses (*n* = 13) have defined environmentally sustainable intensive care as the minimization, conscious separation and recycling of medical waste.

In surgical intensive care units, there is a significant amount of medical waste generated during the postoperative care process. The reduction, separation, and recycling of these medical wastes are crucial for sustainability. (N5)

Reducing the consumption of disposable materials used during patient care is crucial. (N8)

#### 3.1.2 Sub‐theme 1.2: Waste reduction and cost‐effectiveness

More than half of the nurses (*n* = 13) emphasized the reduction of waste as a key aspect of environmentally sustainable intensive care, highlighting that this could contribute to cost‐effectiveness.

Intensive care is a costly place as it is where the latest technological tools, materials and devices are used. Therefore, in order for a high‐cost place to be sustainable, its cost must be minimized, and minimizing the use of energy, medical consumables or other devices and not using them unnecessarily can make intensive care sustainable. (N13)

Reducing unnecessary material consumption in intensive care contributes to reducing the cost to the hospital and contributes to the environmental sustainability of intensive care. (N21)

#### 3.1.3 Sub‐theme 1.3: Leaving a clean environment for the future generations

Some nurses (*n* = 10) have indicated that environmentally sustainable intensive care would contribute to leaving a healthier, cleaner environment for future generations, while some (*n* = 2) have suggested that it could contribute to mitigating global warming.

In order to leave a healthier, cleaner environment to future generations, we can start by preventing waste and reducing the amount of waste in the intensive care unit where we work. (N8)

Waste management, prevention of waste, and recycling contribute environmentally to sustainable intensive care, while also helping mitigate global warming. (N1)

### 3.2 Theme 2: Prevention of waste in intensive care, responsible consumption and recycling

All nurses unanimously emphasized the prevention of waste and the importance of responsible consumption in providing environmentally sustainable intensive care. Recommendations were provided regarding the responsible consumption of medications, energy and consumables commonly used in surgical intensive care.

#### 3.2.1 Sub‐theme 2.1: Drug wastage

Many nurses (*n* = 16) have reported drug wastage in intensive care, attributing it to reasons such as doctors routinely entering the same order without individually checking the computer system, and the pharmacy refusing to accept returned unnecessary medications. Additionally, it was noted that drug waste occurs when prescribed drugs are administered routinely to patients, even when not necessary. To address this issue, it has been suggested that nurses could collaboratively review and make recommendations with doctors during order processing. Emphasis has been placed on the importance of paying attention to the storage conditions of medications and utilizing the existing handheld system to prevent waste.

In particular, we are facing medication waste issues, and I attribute this to factors related to the pharmacy and order processing. Because our doctors do not stop the patient's medicine from the system, even if it is stopped, they continue to give the same order and the medicine comes from the pharmacy. The pharmacy does not take back the unused medications. I believe this issue is primarily doctor‐related. We cannot return medications to the pharmacy; instead, we transfer them to other patients in need. In our hospital information system, we have an inventory control system. After the doctor enters the prescription, the nurse accepts it and can update the inventory with the available medications. We are actually trying to use this system effectively. However, there could be more dedication among the staff in utilizing the inventory control system. (N2)

I try to comply with 8 main points when using medication. It is necessary to prevent loss of dose of drugs, comply with the rules of safe drug use, and pay attention to the transport and storage conditions of drugs. (N13)

#### 3.2.2 Sub‐theme 2.2: Energy waste

The majority of nurses (*n* = 20) have indicated their conscientious efforts to avoid energy waste while working in the ICU. Nurses reported closing unnecessary lights and devices left on. Additionally, they suggested the use of bedside lamps instead of continuously lit lights at night to prevent energy waste, and sensor taps and electric usage should be widespread in order to prevent energy waste.

I believe that having sensor‐operated faucets significantly mitigates water waste, and the implementation of sensor‐operated lights in corridors and washrooms also helps prevent electricity waste. (N9)

The utilization of individual bedside overhead lamps for patients is a potential solution to decrease overall ceiling lighting in the intensive care unit. (N11)

Some nurses have suggested that, during the construction phase of hospitals, the government should review alternative energy sources and implement practices that enhance energy efficiency.

A solar energy‐integrated roofing system can be incorporated into the plans of hospitals. It can at least partially address the electricity demand. The majority of new urban hospitals incur significant expenses in natural gas combustion or air conditioning to mitigate the issue of glass heating. The government should evaluate these projects from an energy‐saving perspective. (N2)

#### 3.2.3 Sub‐theme 2.3: Consumable consumption and recycling

Nearly all nurses (*n* = 20) stated that there was a waste of consumables in the ICU. Additionally, recommendations have been provided concerning medical consumables and personal protective equipment perceived to contribute to the waste issue.

There is a need to develop personal protective equipment suitable for sterilization instead of single‐use items. (N3)

Instead of disposable gowns used in patient care, washable and disinfectable gowns could be considered. This is because, in the current practice, a total of four gowns are used for a single patient when two personnel enter for care twice a day. Extrapolating this to eight patients would result in the use of 32 gowns in a day for a single intensive care unit. (N19)

I believe there is waste occurring in the intensive care unit. To prevent such waste, it is crucial to procure materials of high quality. For instance, injectors and infusion sets are found to be defective, leading to unnecessary waste, and similarly, gloves are discovered to be torn. (N1)

Additionally, it has been recommended to increase the number of recycling bins and place them in easily accessible locations.

Recycling bins can be numerous and easily accessible. (N6)

### 3.3 Theme 3: Suggestions for institutional and individual behavioural change regarding environmental sustainability

All nurses stated that education, reward—encouragement, and control should be provided in order to disseminate practices that ensure environmental sustainability in intensive care, to become institutional culture and to develop behavioural changes in nurses in this regard.

#### 3.3.1 Sub‐theme 3.1: Education

The majority of nurses (*n* = 19) have expressed the need for regular training sessions employing various educational methods to promote environmental sustainability in the ICU.

The lack of sufficient knowledge among relevant individuals in this regard poses a significant obstacle to environmental sustainability. Regular training sessions should be conducted to integrate environmental sustainability into the intensive care unit. (N17)

Providing small‐group, one‐on‐one training sessions, designed to enhance productivity and engagement, rather than mass or online training, will significantly contribute to raising awareness. (N2)

Alongside education, the placement of warning posters can serve as effective reminders. (N14)

#### 3.3.2 Sub‐theme 3.2: Reward—Encouragement

The majority of nurses (*n* = 19) have suggested that in order to promote environmental sustainability and foster behavioural change in the ICU, it would be beneficial to reward and showcase nurses who contribute to environmental sustainability through responsible consumption. They propose conducting competitions at the service level to embrace this culture.

Education alone is not sufficient to bring about behavioural change. The individual must desire it, be incentivized and provided with motivations. Various incentives can be provided, such as selecting intensive care units that produce the least medical waste as a benchmark. This approach has the potential to motivate individuals. Additionally, rewards can be offered. I believe these strategies can effectively contribute to behavioural change. (N15)

Institutions can provide awards and honours to conscientious nurses who demonstrate careful behaviour regarding recycling, aiming to encourage and incentivize environmentally conscious practices. (N22)

The hospital administration or other institutions may select the employee or department of the month. (N1)

#### 3.3.3 Sub‐theme 3.3: Control

The majority of nurses (*n* = 13) have expressed the importance of institutional leaders prioritizing sustainability, monitoring the outcomes of conducted training sessions, tracking the integration of environmental sustainability into the institution and ensuring the provision of necessary facilitations.

First and foremost, the perspectives of the administrative management and responsible individuals need to be assessed on this matter. Unfortunately, if there is a lack of sufficient attention from them, the issue tends to have limited impact and repercussions on hospital staff. (N3)

I believe that if nurse managers in the intensive care unit provide guidance on this matter (without applying pressure), explaining the reasons and managing the situation by sharing their experiences, it can lead to behavioural change. (N11)

After training sessions, administrators should conduct inspections and reward individuals who demonstrate behavioural changes related to environmental sustainability. (N20)

## 4 DISCUSSION

Surgical units engage in a demanding health care service that necessitates expensive equipment, advanced surgical technologies, sterilization processes and life support systems. As a result of these activities, a significant amount of waste is produced, and a significant amount of energy and consumables are used.^24^ ICUs contribute more to greenhouse gas emissions per bed per day than acute care units.^25^ The study was qualitatively conducted to examine the thoughts of nurses working in surgical intensive care services regarding responsible medical waste, energy and medication consumption.

The first theme, ‘Environmentally Sustainable Intensive Care’, was examined. Nurses have defined what environmentally sustainable intensive care means to them. Despite 86.9% of the nurses indicating a lack of knowledge about sustainable development goals, they defined environmentally sustainable intensive care as minimizing medical waste, reducing conscious waste and leaving a clean environment for the future. The majority of participants offering creative solutions despite their lack of knowledge is thought to be associated with implicit knowledge. Similar to our study, another investigation found that nurses were unable to establish a connection between their own roles and sustainable development goals. The study reported a lack of knowledge among nurses about sustainable development goals, indicating that they had not heard of this concept before and highlighting the importance of increasing awareness through education.^26^ In another study, it was indicated that nurses need more information on sustainability to participate in and support actions related to climate change management.^27^ In a study involving nursing students, it was expressed that 63.8% of the students had not heard of the concept of sustainable development before.^28^ The WHO emphasizes the importance of significant investment in nursing education at both undergraduate and postgraduate levels, to further engage and contribute to nurses and the nursing profession with regard to kowledge about sustainable development goals.^29^

In the study, nurses indicated that there is a significant amount of medical waste generated during the postoperative care process in surgical ICUs. This situation is more prominent in patients with surgical site infections, frequent dressing changes and those placed in isolation.^30^ Nurses emphasized the importance of reducing, segregating and recycling the quantity of medical waste for sustainability. Similarly, in the study by McGain et al., medical waste was the major hospital waste.

The importance of initiatives such as waste prevention programmes like Choosing Wisely to reduce the environmental footprint of health care services is emphasized. Simultaneously, avoiding medically unnecessary intensive care admissions and preventing routine opening of disposable items in emergencies are included.^2^ Integrating the ‘5R concept’ (reduce, reuse, recycle, rethink and research) into clinics is indicated to reduce the intensive care carbon footprint and ensure sustainability.^31^ In the study, it has been stated that waste management, waste prevention and recycling will contribute to environmentally sustainable intensive care, aiming to leave a clean environment for the future. Similarly, the study by Baid and colleagues has highlighted the potential benefits in environmental, financial and social aspects because of the integration of sustainability systems, revealing the ripple effect created by these systems.^13^

The second theme of the study is ‘Preventing waste in intensive care, responsible consumption and recycling’. Nurses have indicated that there is a waste of medication and disposable materials in the ICU. They have also emphasized their efforts to avoid energy waste. Nurses, particularly highlighting medication waste, have provided recommendations to prevent waste, emphasizing order control, storage conditions and adherence to usage rules. Similarly, a multicentre study has expressed concern about the alarming dimension of medication waste. It has been noted that preventable medication waste ranges from 7.8% to 85.7%. The study indicated that the total annual waste could vary between 36.1% and 39.7%, with an estimated total cost of $92569.^32^

Nurses have indicated their efforts to avoid energy waste in electricity and water resources while working in the surgical ICU. They mentioned being able to turn off unnecessary energy sources but noted that they lacked the authority to make any interventions in heating and cooling. The health care sector is an institution where water, electricity, natural gas and other resources are significantly consumed. In the study, 91.3% of nurses reported that electricity is the most consumed energy source in intensive care. This was followed by 17.3% for water and 4.3% for natural gas. In a study examining a hospital's energy consumption, the daily consumption per bed in the ICU was found to be approximately 84 kWh of electricity, 258 MJ of natural gas and 0.7 m^3^ of water. The study reported that 25%–33% of greenhouse gas emissions in the intensive care unit are attributed to energy usage. It has been reported that an estimated 121 kg CO_2_e is produced per bed per day in the ICU.^33^

In the sustainability strategy of ICUs, there should be a vision to reduce health care carbon emissions, conserve energy resources, and support healthy lifestyles and the environment. In our study, the most consumed disposable items were reported as medical supplies such as injectors, intravenous catheter, needle tips, ranking first at 73.9%, followed by dressing materials, personal protective equipment, catheters, bed linens and paper, respectively. In the study by Sürme and Maraş, nurses mentioned the frequent use of gloves, injectors, cannulas, needles, etc. as the most used materials in the ward.^7^ In the ICU, it has been noted that the most consumed materials include gowns, catheters, gloves, EKG electrodes and aspiration materials. The daily cost per bed for the consumption of these materials is reported to be $31.^33^

Life cycle analyses (LCD) reveal that pharmaceuticals and medical devices significantly contribute to CO_2_ emissions in health care. Effective management of material and drug stock levels is recommended to reduce waste. Reusable products provide savings compared to disposable ones. If reduction or reuse is not possible, recycling methods should be employed.^2^ Nurses, being the largest group providing services in health care institutions, are the primary users of resources and equipment in ICUs. Increasing awareness among nurses about waste reduction in resource usage is an important and effective factor.^19^

The third theme is ‘Suggestions for institutional and individual behavioural change regarding environmental sustainability’. Nurses emphasized the need for education, reward—encouragement, and control to instil environmental sustainability behaviour. Similarly, in Trent et al.'s study, creating widespread awareness for a sustainable ‘green team’ was recommended through regular announcements in clinical meetings, boards and social media platforms. Their study highlights the importance of celebrating, promoting and incentivizing successes, as well as fostering idea and team collaboration similar to our study.^25^ In the study by Martin and colleagues, the ‘green team’ established in the ICU has systematically sorted and regulated the reuse of waste in the intensive care room from the moment it is generated. By the end of the study, the total waste amount was reduced from 58.9% to 29.9%. Additionally, 651 kg of plastic and 4808 kg of cardboard were recycled.^34^

Understanding how nurses' bedside actions contribute negatively to climate change, from the disposal of the products they use to recycling, and being aware of how much electricity and water they use in patient care is important. It is essential for them to relate this awareness to climate change and sustainable development goals. Health institutions also need to take an active role in reducing the impacts of climate change across their organizations.

### 4.1 Recommendations or implications for practice and/or further research

Surgical ICUs are high‐resource, high‐energy consumption and high‐waste production units because of their multifunctional activities. It is important to conduct various studies in these units, where the impact on greenhouse gas emissions has not been clearly elucidated. Sustainability strategies should be created in institutions to ensure responsible medical waste management, energy and medicine consumption and reduce carbon footprint. In accordance with this purpose, ‘Green teams’ including unit‐based doctors, nurses and paramedics should be established. Training should be provided and awareness should be raised to reduce energy use resulting from heating, lighting, ventilation and air conditioning. Life cycle assessment should be conducted for reusable and recyclable materials and drugs. Taking economically, ecologically or socially sustainable steps through collaboration between health care professionals and institutions is crucial for green hospitals and ICUs.

### 4.2 Limitations

Our study has some limitations. This study's limitations include how the semi‐structured interview focused on particular questions and how more detailed and comprehensive information could be gathered by including a wider range of intensive care environmental sustainability topics. The research was conducted in surgical ICUs of one hospital in Turkey; therefore, further qualitative and quantitative researches across various countries and types of ICUs would help develop a stronger evidence base.

## 5 CONCLUSIONS

As a result of the study, it was reported that the most frequently used consumables in intensive care were injectors, intravenous catheters and needle tips, and electricity was among the most used energy sources. Most nurses emphasized the lack of awareness and education about sustainable development goals. Nurses defined environmentally sustainable intensive care as minimizing medical waste, conscious separation and recycling, reducing waste and cost‐effectiveness, and leaving a clean environment for future generations. Nurses emphasized the importance of waste prevention and responsible consumption in providing environmentally sustainable intensive care. Recommendations were made regarding the drug and energy waste and consumables commonly used in surgical intensive care. Nurses stated that education, reward—encouragement, and control should be provided to disseminate intensive sustainability practices, make them a corporate culture and develop behavioural changes in nurses in this regard.

## AUTHOR CONTRIBUTIONS

YS and GM contributed to conception, design, critical revision and writing of the article, and provided final approval for the manuscript. YS, GM and GAA contributed to review, critical revision and writing of the article, and provided final approval for the manuscript.

## Data Availability

The data that support the findings of this study are available on request from the corresponding author. The data are not publicly available due to privacy or ethical restrictions.

## References

[1] Molero A , Calabrò M , Vignes M , Gouge GD . Sustainability in healthcare: perspectives and reflections regarding laboratory medicine. Anna Lab Med. 2021;41(2):139‐144. doi:10.3343/alm.2021.41.2.139 PMC7591295 33063675

[2] McGain F , Muret J , Lawson C , Sherman JD . Environmental sustainability in anaesthesia and critical care. Br J Anaesth. 2020;125(5):680‐692. doi:10.1016/j.bja.2020.06.055 32798068 PMC7421303

[3] Tennison I , Roschnik S , Ashby B , et al. Health care's response to climate change: a carbon footprint assessment of the NHS in England. Lancet Planetary Health. 2021;5(2):84‐92. doi:10.1016/S2542-5196(20)30271-0 PMC7887664 33581070

[4] Eckelman Matthew J , Huang K , Lagasse R , Senay E , Dubrow R , Sherman JD . Health care pollution and public health damage in the United States: an update. Health Aff. 2020;39(12):2071‐2079. doi:10.1377/hlthaff.2020.01247 33284703

[5] Bein T , McGain F . Climate responsibilities in intensive care medicine—let's go green! An introduction to a new series in intensive care medicine. Intensive Care Med. 2023;49(1):62‐64. doi:10.1007/s00134-022-06930-8 36446855 PMC9852098

[6] Keil M . The greenhouse gas emissions of a German hospital‐a case study of an easy‐to‐use approach based on financial data. Cleaner Environ Syst. 2023;11:100140. doi:10.1016/j.cesys.2023.100140

[7] Mansur F , Korkmaz S . Sağlık hizmeti kullanıcılarının yeşil hastane farkındalık düzeylerini belirlemeye yönelik bir çalışma. Ankara Hacı Bayram Veli Üniversitesi İktisadi Ve İdari Bilimler Fakültesi Dergisi. 2020;22(3):827‐850.

[8] Hunfeld N , Diehl JC , Timmermann M , et al. Circular material flow in the intensive care unit—environmental effects and identification of hotspots. Intensive Care Med. 2023;49(1):65‐74. doi:10.1007/s00134-022-06940-6 36480046 PMC9734529

[9] World Health Orgazination . Healthy Hospitals Healthy Planet Healthy People, Addressing climate change in health care settings. 2009. https://www.who.int/docs/default-source/climate-change/healthy-hospitals-healthy-planet-healthy-people.pdf?sfvrsn=8b337cee_1

[10] International Council of Nurses . Position Statement: Nurses, Climate Change and Health. International Council of Nurses; 2008. https://www.icn.ch/sites/default/files/inline-files/ICN%20PS%20Nurses%252c%20climate%20change%20and%20health%20FINAL%20.pdf

[11] Wohlford S , Nathalia EF , Kimberly FC . Reducing waste in the clinical setting. Am J Nurs. 2020;120(6):48‐55. doi:10.1097/01.NAJ.0000668744.36106.24 32443125

[12] İlaslan N , Çakar M . 2030 sürdürülebilir kalkınma hedefleri kapsamında gezegen sağlığı ve gezegen hemşireliğinin önemi. Türkiye Klinikleri Hemsirelik Bilimleri. 2021;13(3):717–724. doi: 10.5336/nurses.2020‐79658

[13] Baid H , Richardson J , Scholes J , Hebron C . Sustainability in critical care practice: a grounded theory study. Nurs Crit Care. 2021;26(1):20‐27. doi:10.1111/nicc.12493 31828900

[14] Kim H , Sefcik JS , Bradway C . Characteristics of qualitative descriptive studies: a systematic review. Res Nurs Health. 2017;40(1):23‐42. doi:10.1002/nur.21768 27686751 PMC5225027

[15] Hall S , Liebenberg L . Qualitative description as an introductory method to qualitative research for Master's‐level students and research trainees. Int J Qual Methods. 2024;23:16094069241242264. doi:10.1177/16094069241242264

[16] Sullivan‐Bolyai S , Bova C . Qualitative Description: A How‐to Guide. Graduate School of Nursing, University of Massachusetts Medical School; 2021. doi:10.13028/8vwe-xc61

[17] The Ministry of Health of Türkiye Health Statistics Year Book. 2022. https://dosyasb.saglik.gov.tr/Eklenti/48055/0/siy2022eng050420241pdf.pdf

[18] Kyngäs H , Mikkonen K , Kääriäinen M . The Application of Content Analysis in Nursing Science Research. Springer; 2020.

[19] Sürme Y , Maraş G . Recycling, responsible consumption and nursing: a qualitative study of surgical nurses' recycling and medical waste management. J Nurs Manag. 2022;30(8):4514‐4522. doi:10.1111/jonm.13891 36326215

[20] Colaizzi PF . Psychological research as the phenomenologist views it. In: Valle R , King M , eds. Existential Phenomenological Alterations for Psychology. Oxford University Press; 1978:48‐71.

[21] Tong A , Sainsbury P , Craig J . Consolidated criteria for report‐ing qualitative research (COREQ): a 32‐item checklist for interviewsand focus groups. International J Qual Health Care. 2007;19(6):349‐357. doi:10.1093/intqhc/mzm042 17872937

[22] Colorafi KJ , Evans B . Qualitative descriptive methods in health science research. Herd. 2016;9(4):16‐25. doi:10.1177/1937586715614171 PMC7586301 26791375

[23] Probst B . The eye regards itself: benefits and challenges of reflex‐ivity in qualitative social work research. Soc Work Res. 2015;39(1):37‐48. doi:10.1093/swr/svu028

[24] MacNeill AJ , Lillywhite R , Brown CJ . The impact of surgery on global climate: a carbon footprinting study of operating theatres in three health systems. Lancet Planetary Health. 2017;1(9):e381‐e388. doi:10.1016/S2542-5196(17)30162-6 29851650

[25] Trent L , Law J , Grimaldi D . Create intensive care green teams, there is no time to waste. Intensive Care Med. 2023;49(4):440‐443. doi:10.1007/s00134-023-07015-w 36964216 PMC10038378

[26] Fields L , Perkiss S , Dean BA , Moroney T . Nursing and the sustainable development goals: a scoping review. J Nurs Scholarsh. 2021;53(5):568‐577. doi:10.1111/jnu.12675 34056841

[27] AnAaker A , Nilsson M , Holmner A , Elf M . Nurses' perceptions of climate and environmental issues: a qualitative study. J Adv Nurs. 2015;71(8):1883‐1891. doi:10.1111/jan.12655 25810044

[28] Şimşek HG , Erkin Ö . Sustainable development awareness and related factors in nursing students: a correlational descriptive study. Nurse Educ Pract. 2022;64:103420. doi:10.1016/j.nepr.2022.103420 35952472

[29] World Health Organization . State of the world's nursing 2020: Investing in education, jobs and leadership. 2020. https://www.who.int/publications/i/item/9789240003279

[30] Anstey MH , Trent L , Bhonagiri D , et al. How much do we throw away in the intensive care unit? An observational point prevalence study of Australian and New Zealand ICUs. Crit Care Resusc. 2023;25(2):78‐83. doi:10.1016/j.ccrj.2023.05.004 37876601 PMC10581268

[31] Schuster M , Richter H , Pecher S , Koch S , Coburn M . Ecological sustainability in anesthesiology and intensive care medicine. A DGAI and BDA position paper with specific recommendations. Anaesth Intensivmed. 2020;61:329‐338. doi:10.19224/ai2020.329

[32] Barbariol F , Deana C , Lucchese F , et al. Evaluation of drug wastage in the operating rooms and intensive care units of a regional health service. Anesth Analg. 2021;132(5):1450‐1456. doi:10.1213/ANE.0000000000005457 33667211

[33] Prasad PA , Joshi D , Lighter J , et al. Environmental footprint of regular and intensive inpatient care in a large US hospital. Int J Life Cycle Assess. 2022;27:38‐49. doi:10.1007/s11367-021-01998-8

[34] Martin C , Minville V , Mayeur J , Conil JM , Vardon‐Bounes F . Transforming waste management in intensive care units: a path towards environmental sustainability and resource optimization. Intensive Care Med. 2023;49(9):1136‐1137. doi:10.1007/s00134-023-07091-y 37439873

